# Scorpins in the DNA Damage Response

**DOI:** 10.3390/ijms19061794

**Published:** 2018-06-17

**Authors:** Dario Palmieri, Anna Tessari, Vincenzo Coppola

**Affiliations:** Department of Cancer Biology and Genetics, College of Medicine, The Ohio State University and James Comprehensive Cancer Center, Columbus, OH 43210, USA; dario.palmieri@osumc.edu (D.P.); Anna.tessari@osumc.edu (A.T.)

**Keywords:** RANBP9, RANBPM, RANBP10, Scorpins, DDR, GID complex, CTLH complex

## Abstract

The DNA Damage Response (DDR) is a complex signaling network that comes into play when cells experience genotoxic stress. Upon DNA damage, cellular signaling pathways are rewired to slow down cell cycle progression and allow recovery. However, when the damage is beyond repair, cells activate complex and still not fully understood mechanisms, leading to a complete proliferative arrest or cell death. Several conventional and novel anti-neoplastic treatments rely on causing DNA damage or on the inhibition of the DDR in cancer cells. However, the identification of molecular determinants directing cancer cells toward recovery or death upon DNA damage is still far from complete, and it is object of intense investigation. *S*PRY-*co*ntaining *R*AN binding *P*rote*ins* (*Scorpins*) RANBP9 and RANBP10 are evolutionarily conserved and ubiquitously expressed proteins whose biological functions are still debated. RANBP9 has been previously implicated in cell proliferation, survival, apoptosis and migration. Recent studies also showed that RANBP9 is involved in the Ataxia Telangiectasia Mutated (ATM) signaling upon DNA damage. Accordingly, cells lacking RANBP9 show increased sensitivity to genotoxic treatment. Although there is no published evidence, extensive protein similarities suggest that RANBP10 might have partially overlapping functions with RANBP9. Like RANBP9, RANBP10 bears sites putative target of PIK-kinases and high throughput studies found RANBP10 to be phosphorylated following genotoxic stress. Therefore, this second Scorpin might be another overlooked player of the DDR alone or in combination with RANBP9. This review focuses on the relatively unknown role played by RANBP9 and RANBP10 in responding to genotoxic stress.

## 1. Introduction

The DNA damage response (DDR) is an attractive target for anti-cancer treatments due to the increased genotoxic stress that malignant cells experience [1,2]. Modalities of repair of the damage are limited to a handful of recognized mechanisms (reviewed in [3]). In cancer cells, one or more of these mechanisms are deficient or altered, providing an opportunity of achieving synthetic lethality by inhibition of the remaining ones [4]. For example, BRCA-deficient malignancies are exquisitely sensitive to Poly(ADP)-Ribose Polymerase (PARP) inhibition [2,4,5]. Nonetheless, mounting evidence shows that cells adapt. They find ways to reactivate targeted/inactivated proteins or resort to alternative pathways guarantying DNA repair at levels compatible with survival and proliferation [2,5]. For these reasons, finding vulnerabilities that are specific to cancer cells sparing normal ones has proven to be challenging [2,5]. In addition, incomplete understanding of the network and missing tiles of the complex puzzle represented by unrecognized players are main reasons of the failure of anticancer treatments aiming to target DNA repair mechanisms [2,4,6].

*S*PRY-*co*ntaining *R*AN binding *P*rote*ins* (*Scorpins*) RANBP9 and RANBP10 are two highly similar proteins whose biological functions are poorly understood. This inadequate knowledge is due to multiple causes including the lack of validated antibodies able to discriminate between the two proteins. Moreover, the perinatal lethality of RANBP9 knockout mice has also negatively affected the investigation of its biological functions.

Comprehensive reviews about proposed biological roles of RANBP9 in cancer [7], Alzheimer’s disease and other contexts [8,9,10] are available. On the other hand, there are no exhaustive reviews about RANBP10 but only a few studies about its role in platelet physiology [11,12,13].

This review focuses on the relatively unknown role of Scorpins in responding to genotoxic stress.

## 2. Scorpins

RAN Binding Protein 9 (RANBP9) is a ~90 Kd scaffold protein that due to “*a series of unfortunate events*” has been labeled and probably investigated from the wrong perspective through the years. One of its first names was B-cell antigen receptor Ig Beta-Associated Protein 1, (IBAP-1) even if there is no evidence supporting any molecular role of this protein in B-cell signaling or the immune system in general (NCBI Gene ID: 56705). Another commonly used name is RANBPM (RAN Binding Protein Microtubule organizing center), which derives from the initial observation that the protein localized at the Microtubule Organizing Center (MTOC) [14]. However, a subsequent article from the same authors reported that they had been using only a truncated form of the wild type protein, lacking 229 amino acids at the *N*-terminus. Thus, they refuted their previous conclusion and showed that the full-length RANBP9 does not localize to the MTOC [15]. Finally, in the absence of a better functional characterization, the current preferred name of this scaffold protein is RAN Binding Protein 9 due to sequence homology with the RAN binding domain present on other proteins [14]. However, the evidence supporting a binding of RAN by RANBP9 is limited to the initial publication twenty years ago, was never reconfirmed and it is very weak at best [14]. For this reason, whether RANBP9 really binds and affects RAN functions remains to be formally proven and better investigated.

RAN Binding Protein 10 (RANBP10) was discovered as a paralog of RANBP9 and was given a name with the next numeral [16]. It has been shown in megakaryocytes that RANBP10 binds RAN and acts as a *bona fide* RAN-GTP Exchange Factor (GEF) in the cytoplasm although it showed a substantial lower specific activity than RCC1 (Regulator of Chromosome Condensation 1) RAN-GEF used as control in those experiments [11].

**Figure 1:**
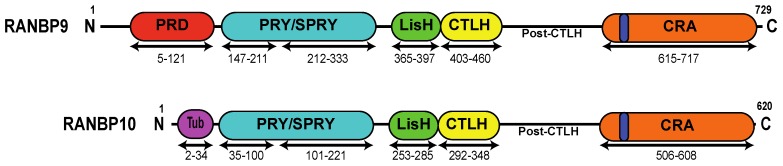
Schematic representation of RANBP9 and RANBP10 proteins. RANBP10 shares high amino acid conservation with RANBP9 in the PRY (94%), SPRY (97%), LisH (82%), CTLH (90%), and CRA (89%) domains. The two proteins differ the most at the N-terminus and in the post-CTLH region, which contains several putative PIK-kinase phosphorylation sites (see Table 1). PRD (red)= Proline-Rich Domain; PRY/SPRY (light blue) = Spore lysis A and Ryanodine receptor Domain; LisH (green) = Lissencephaly type-I-like homology motif; CTLH (yellow) = Carboxy-terminal to LisH motif domain; CRA (orange) = CT11-RanBP9 domain; dark blue = putative Nuclear Localization Signal; Tub (purple) = tubulin-binding domain.

Because of their initial identification as RAN binding proteins, it was assumed that Scorpins participate in the nuclear-cytoplasmic import-export process [14,17]. In some databases RANBP9 is still confused with Importin 9, which is a distinct protein of 1048 amino acids (UniProtKB-Q96P70). While it is established that RANBP9 and RANBP10 are present both in the cell nucleus and cytoplasm, there is no evidence of a role of these two proteins in nuclear import/export. On the contrary, RANBP9 and RANBP10 are structurally very different from other RAN binding proteins (Figure 1). They bear four known domains and based on the distinctive presence of a PRY/SPRY domain they should more appropriately be called Scorpins, forming a separate subgroup by themselves as initially suggested by Hosono et al. [18]. For more information about the structure and the putative functions not related to the DDR of the different domains of Scorpins we recommend the reading of earlier reviews [7,8,10,19,20].

## 3. Known Biological Roles of RANBP9

RANBP9 is a ubiquitous scaffold protein present both in the cell nucleus and cytoplasm. It is evolutionarily highly conserved and its functions are poorly characterized [7,8]. The generation of *RANBP9* knockout (KO) mice demonstrated that this protein is not required for embryonic development. However, most of *RANBP9* KO animals die immediately after birth possibly because of lactation failure. The few survivors are smaller in size compared to their littermates and completely lack spermatogenesis or oogenesis [21]. Of note, RANBP9 has been linked to different types of cellular stress and might work as part of macromolecular complexes [7].

**Figure 2:**
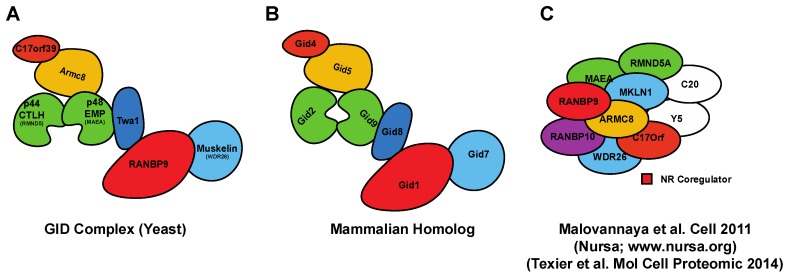
Schematic representation of the *S. cerevisiae* Glucose-Induced degradation Deficient (GID)- and correspondent mammalian CTLH-macromolecular complexes. (**A**) The topology of the GID complex in yeast is well established [31]; (**B**) Predicted composition of the mammalian CTLH complex based on the GID mammalian homologs. The name of the complex comes from the CTLH domain that most of the members have; (**C**) CTLH or Nuclear Receptor coregulator-complex pulled down from mammalian cells including RANBP9 and RANBP10 (adapted from [29]). In the depicted complex, GID8 is named C20orf11 and indicated as C20. The experiment showed as part of the complex also YPEL5 (Yippee Like 5) indicated as Y5 in the cartoon, which has no known equivalent in the *S. cerevisiae* GID complex.

RANBP9 has been reported to directly or indirectly interact with more than a hundred proteins [7]. The *Genecards* database lists about 400 potential interactions (www.genecards.org). It is striking that the putative partners reported for RANBP9 do not share any significant structural homology or belong to one particular group of protein or cellular pathway [7,8]. On the contrary, RANBP9 putative interactors are very different among each other and participate in a wide variety of biological processes [7,8]. Many of the published interactions are derived from yeast-two-hybrid experiments or limited to co-immunoprecipitations performed upon exogenous over-expression of both RANBP9 and/or its putative partners. A good number of these interactions was never independently confirmed in subsequent studies and thus needs to be taken with caution. In several instances, the presence or absence of RANBP9 has been shown to have an effect on the amount of the interacting partner [22,23,24,25,26,27]. For example, RANBP9 has been shown to prevent the turnover of the mammalian homolog of the Lethal giant larvae (Mgl-1) tumor suppressor by facilitating the de-ubiquitination of the protein mediated by USP11 [23,28]. Also, relevant to the DDR, RANBP9 was shown to stabilize p73α [22]. However, how RANBP9 affects protein stability remains to be elucidated. In this regard, one potential mechanism of action in regulating protein turnover is through its role as corner stone necessary to assemble the CTLH complex [7,29,30], which is the mammalian equivalent of the Glucose-Induced degradation Deficient (GID) complex in yeast ([31,32] and Figure 2). The *S. cerevisiae* homolog of RANBP9 is GID1 and it is necessary for the formation of the entire complex, a putative E3 ligase macromolecular structure that degrades metabolic enzymes no longer needed after nutrient replenishment [31,32]. GID1 is also named VID30 (Vacuole-Induced degradation Deficient 30) because it can mediate the degradation of the same metabolic enzymes via lysosome [33,34]. Therefore, it is tempting to speculate that RANBP9 might regulate lysosome-mediated protein degradation. However, clear evidence supporting GID- or VID-like functions in mammalian cells is still lacking.

## 4. RANBP9 in the DDR

Several studies have linked RANBP9 to cellular response to multiple types of stress, including DNA damages upon exposure to ionizing radiation or chemotherapeutic drugs.

### 4.1. RANBP9 and Sensitivity to Ionizing Radiation (IR)

One of the most interesting unbiased studies was performed by Yard and colleagues, where 533 genetically annotated human cancer cell lines were analyzed by large scale profiling after exposure to ionizing radiation. This study identified RANBP9 as one of the top 19 genes associated with radiation sensitivity when mutated [35].

### 4.2. RANBP9 and Post-Translational Modifications Following Stress

Genotoxic stress caused by cisplatin, Ultra Violet (UV)-light, osmotic shock and Ionizing Radiation (IR) cell exposure [26,36] leads to RANBP9 phosphorylation by kinases that have been identified only to a limited extent. A list of predicted and experimentally proven Serine or Threonine phosphorylation target sites of PIK-kinases is shown in Table 1. However, other RANBP9 residues are predicted to be phosphorylated by other kinases that are not immediately relevant to the DDR and thus left out from our review. At least in part, specific post-translational modifications have been shown to influence both the sub-cellular localization of RANBP9 and its ability to bind different molecular partners, potentially mediating its biological functions [37].

### 4.3. RANBP9 Protein-Protein Interactions Relevant to the DDR

Several notable RANBP9 interactions reported in literature are with factors directly or indirectly associated to cellular DNA damage response [7]. These interactions might contribute to explain some of the phenotypes observed in the context of genotoxic stress when RANBP9 levels are altered.

Most of the reported RANBP9 interactions point to a potential involvement in determining apoptotic cell death by regulating mitochondrial function and membrane permeability, as described below. However, recent studies have demonstrated that many proposed RANBP9-interacting proteins in the nucleus can modulate cell ability to arrest cell cycle progression and affect repair of DNA upon different types of damages.

For example, RANBP9 over-expression has been shown to enhance dephosphorylation of the primary non-muscle isoform of ADF (Actin Depolymerizing Factor)/Cofilin (CFL-1) [38,39]. This event results in CFL-1 translocation to the mitochondria and promotes apoptosis [40,41]. However, it has been also demonstrated that overexpression of wild-type (phosphorylatable) CFL-1 can affect DNA repair capacity and results in cell death due to accumulation of damaged DNA. In fact, CFL-1 may localize into the nucleus preventing ATM-dependent phosphorylation of H2AX and recruitment of critical mediators of Homologous Dependent Repair (HDR) of DNA Double Strand Breaks (DSBs) [25,38,39,40,42,43,44,45]. Therefore, we can speculate that a decrease or complete ablation of RANBP9 might result in an increase of nuclear phosphorylated CFL1, which, in turn, causes a blunting of the ATM activation and DNA repair by HDR. Interestingly, the latter is in agreement with our group’s observations in lung cancer cells in which RANBP9 was silenced [26].

Wang and colleagues described the nuclear interaction between RANBP9 and Homeodomain Interacting Protein Kinase 2 (HIPK2) [46], a known mediator of apoptosis upon interaction with the death receptor Tumor Necrosis Factor (TNF) receptor type 1-associated death domain protein [46]. Notably, HIPK2 is activated upon phosphorylation by ATM and it controls DNA-damage dependent cell fate through the phosphorylation of key players of the DDR, such as p53 and the anti-apoptotic co-repressor C-terminal binding protein (CtBP) [47].

In a model of Alzheimer’s disease, Liu et al. demonstrated that RANBP9 physically associates with the tumor suppressor p73, enhancing its level both transcriptionally and post-transcriptionally, cooperating as a complex mediating mitochondria-dependent apoptosis [22,48]. Notably, p73 is a critical transcriptional regulator of genes involved in DNA repair, such as BRCA2, Rad51 and mre11 [49].

Of extreme interest from a DDR perspective is the reported interaction between Ubiquitin Specific Protease 11 (USP11) and RANBP9, which was considered responsible of the ubiquitination and degradation of the tumor suppressor Mgl1 [23,28]. However, recent studies have identified USP11-dependent p21 de-ubiquitination (which results in enhanced G2/M cell cycle checkpoint) [50] and regulation of the PALB2-BRCA1-BRCA2 interaction, required for the activation of HDR DNA repair specifically in the G2/M (and not in the G1) phase of the cell cycle [51]. Therefore, we can speculate that RANBP9 might influence USP11 functions in response to DNA damaging agents. It would be interesting to investigate whether RANBP9 nuclear accumulation during early phases of the DDR [26] has any effect on the PALB2-BRCA1-BRCA2 interaction.

Finally, both RANBP9 and RANBP10 interact with the Androgen Receptor (AR) and have been involved in regulation of AR-mediated transcription [52,53]. AR has been implicated in the DDR [54], particularly because of its ability to regulate the expression of multiple DNA repair genes (e.g., PARP1, FANCI, FANCC, MRE11A, ATR and NBN) involved in different steps of the DDR [55]. We can speculate that the nuclear accumulation during early phases of the DDR [26] might increase transcription of AR-dependent DDR genes. On the contrary, RANBP9 ablation might negatively impact this mechanism.

It is clear that a deeper investigation of RANBP9 interactions with recognized players of the DDR will be required to shed the light on novel mechanisms through which RANBP9 contributes to the response to genotoxic stress.

### 4.4. RANBP9 as a Target and Signaling Facilitator of the Ataxia Telangiectasia Mutated (ATM) Kinase

**Figure 3:**
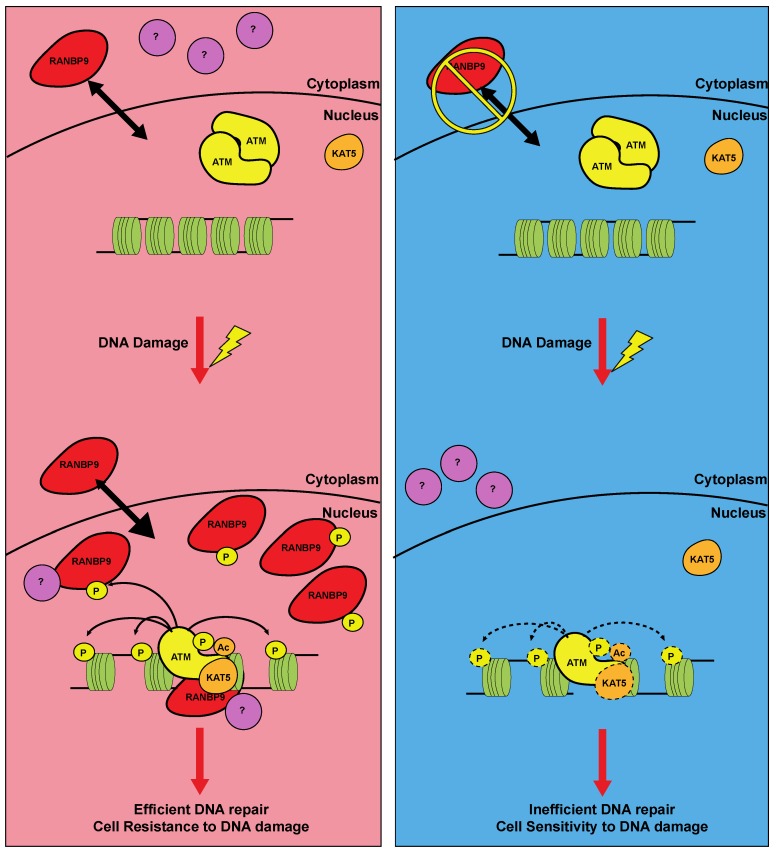
Potential action mechanism of RANBP9 in ATM-dependent DDR. In RANBP9 (red) expressing cells and in the absence of DNA damage (left panel, top), RANBP9 protein shuttles between the nucleus and the cytoplasm. Upon DNA damage such as IR and DNA-damaging drugs (left panel, bottom), ATM is activated and enhances RANBP9 nuclear accumulation through its phosphorylation, potentially with other cytoplasmic partners (purple). This event potentially leads to enhanced KAT5-dependent ATM acetylation, a marker of its full activation. For this reason, RANBP9-expressing cells activate an efficient ATM signaling pathway, resulting in efficient DNA repair and survival to genotoxic stress. Conversely, when RANBP9 expression is reduced, the full activation of ATM is impaired, leading to inefficient DNA repair and sensitivity to DNA damaging agents.

Our group has recently reported that, in response to IR, RANBP9 is phosphorylated on at least 3 different residues (S181, S550, and S603) by ATM, one of the most important kinases involved in the HDR of DNA DSBs. Upon ATM activation and phosphorylation, RANBP9 rapidly (2–8 h) accumulates in the nucleus and participates to the full activation of ATM, through a mechanism still not completely understood. Our results indicated that, in the absence of RANBP9, ATM acetylation, a post-translational modification linked to its complete activation [56,57,58,59,60], is reduced. This observation led us to hypothesize a model (Figure 3) in which, after DNA damage, RANBP9 is promptly recruited and accumulated into the nucleus upon ATM activation, to participate to its full activation, efficient DNA repair and cell survival. Conversely, when RANBP9 levels are abrogated, full activation of ATM and its downstream pathway are impaired, resulting in inefficient DNA repair and, eventually, cell death.

Several different questions remain open for this model, particularly how RANBP9 participates to ATM acetylation and full activation of ATM. A potential candidate for this molecular event is the histone acetyl-transferase (HAT) Tip60 (also know as KAT5), previously reported as RANBP9 interactor in the regulation of AICD transcriptional activity [61]. Interestingly, c-Abl-dependent Tip60 phosphorylation in response to DNA damage enhances its DNA binding activity and acetylation of ATM [56,57,58,59,60,62]. Further studies will be required to demonstrate the existence of a RANBP9/Tip60/ATM pathway required for the complete activation of DNA damage response.

Along the same lines, it is conceivable that, upon its quick nuclear accumulation, RANBP9 might attract into the nucleus additional factors directly or indirectly participating in repair of damaged DNA. However, since the protein is normally present in the nucleus, the currently available tools and reagents do not allow establishing whether RANBP9 is localized at foci of DNA damage.

Another interesting open question that will require further investigation is whether the re-localization of RANBP9 is in the cytoplasm at later (24–72 h) time points [7,37], is associated to the resolution of the DNA damage response and/or to the activation of cell death.

### 4.5. RANBP9 as Pro-Apoptotic Tumor Suppressor

Our group has found that the ablation of RANBP9 in lung cancer cells renders them more sensitive to ionizing radiation and cisplatin exposure. In addition, when RANBP9 is silenced, the homology-directed DNA Repair mechanism is affected, dropping to about 50% of the controls cells [26]. However, earlier studies provide data that are in contrast with our findings [42,43,48,63]. For example, the Kang’s lab has shown that RANBP9 cooperates with p73 to induce apoptosis. Over-expression of RANBP9 in neuronal cells alters the Bax/Bcl2 ratio, promotes Bax oligomerization and induces Cytochrome C from the mitochondria. Simultaneously, mitochondrial fragmentation is observed [42,43,48]. These observations were made in neuronal cells without testing the sensitivity to DNA damaging agents.

The Schild-Poulter lab has provided additional evidence for a pro-apoptotic tumor suppressive role of RANBP9 [63]. Using HeLa cells, they have shown that upon exposure to ionizing radiation RANBP9 accumulates into the cytoplasm at times longer than 24 hours. However, they did not study the dynamics of RANBP9 accumulation at earlier time points. In addition to showing that over-expression of RANBP9 causes cell death, they also showed that HeLa cells in which RANBP9 was silenced are less sensitive to ionizing radiation in direct contrast with our observations. In addition, they show that when RANBP9 was absent levels of Bcl2 are increased in the cytoplasm and less Bax is present at the mitochondria [63].

While it is conceivable that the different cellular models and conditions might explain the contrasting results, it is also important to note that RANBP9 (and RANBP10) are factors involved in maintaining cellular homeostasis and working in concert with other proteins. Therefore, some of the results obtained by forced over-expression or silencing of RANBP9 might have the same deleterious end-result on cellular fitness.

In summary, based on a growing body of evidence, it is conceivable that RANBP9 exerts multifaceted effects in DDR depending on multiple factors like the type of genotoxic stress, the cellular context, and its molecular sub-cellular localization.

## 5. Known Biological Roles of RANBP10

RANBP10 is the least studied of the two Scorpins. The “lack of interest” in this protein is at least in part due to the absence of an obvious phenotype of the RANBP10 knockout mouse. In fact, genetic inactivation of RANBP10 in mouse does not cause early post-natal lethality or infertility like reported for RANBP9 [21,64]. Specialized studies established that RANBP10 knockout mice have a mild platelet deficit due to microtubule functional anomalies [13,65]. Furthermore, RANBP10 is expressed at lower levels compared to RANBP9 and it was shown that RANBP10 not only was not able to increase activation of the RAS/ERK pathway like its paralog, but was competing for the binding of cMET at the membrane and inhibiting the kinase signaling mediated by RANBP9 [16]. However, in addition to the significant structural similarities and the overlapping ubiquitous expression with RANBP9, RANBP10 was pulled-down as component of the CTLH complex similarly to its paralog ([29,30] and Figure 2C). Furthermore, the two proteins were co-immunoprecipitated and shown to have an overlapping cellular distribution by immunofluorescence [53]. In the *Genecards* database and excluding the interaction with RANBP9, there is currently a list of 87 proteins putatively interacting with RANBP10. Only 15 of those potential interactions are not shared with its paralog. Therefore, it is conceivable that RANBP9 and RANBP10 might have overlapping functions and is able to compensate for each other in physiological conditions or in their collective roles in the DDR.

## 6. RANBP10 in the DDR

Based on the hypothesis that Scorpins may have overlapping functions, the role of RANBP10 in the DDR needs to be elucidated. Up-to-date, there are no studies linking directly RANBP10 to the DDR or showing that RANBP10 ablation has an effect on the DDR signaling network as we have shown for RANBP9. An analysis of the RANBP10 amino acidic sequence reveals the presence of several sites putative target of PIK kinases (Table 1). In particular, S69 within the PRY domain is included in a long stretch (51 amino acids) that is highly similar to the region on RANBP9, where it corresponds to ATM-phosphorylated S181. We have previously demonstrated that ATM phosphorylates RANBP9 S181 in vitro using a peptide that is present in both Scorpins ([26] and Table 1). Therefore, we can speculate that RANBP10 S69 is a direct target of ATM too.

RANBP10 was not identified as a target of ATM or ATR in the seminal study by Matsuoka et al., in which they found RANBP9 S603 phosphorylated by ATM [66]. However, subsequent high throughput studies comparing proteomics before and after genotoxic stress list RANBP10 as protein subject to post-translational modifications. For example, S361, S365, and S369 are phosphorylated following ionizing radiation. The same data set reports only RANBP9 S477 as phosphorylated [67]. In Pines et al. (2011), residues S363, S389, S397 are reported as phosphorylated [68]. In Elia et al. (2015), RANBP10 K293 is acetylated following UV [69]. While these reported post-translational modification following genotoxic stress does not constitute compelling evidence, they nevertheless suggest that RANBP10 is involved in the DDR. Also, some of these reported sites are not predicted by our analysis in Table 1. Therefore, future in depth investigations will be required to establish their relevance in the context of the DDR.

## 7. Future Perspectives

Scorpins are poorly studied in general. Therefore, there are many open questions that need to be answered to clarify their biological roles and their involvement in the DDR specifically. One immediate question that can be addressed and prioritized is whether the ablation of RANBP10 alone or in combination with RANBP9 affects the sensitivity to genotoxic stress. Experiments inducing DNA damage in RANBP10 KO cells or mice are not reported. Due to the clinical implications, it is important to establish if the absence of RANBP10 sensitize cancer cells to ionizing radiation or genotoxic drugs in general in a similar manner to what we reported for RANBP9. We do not yet know whether RANBP10 behave similarly in cells following DNA damage. Most importantly, it is necessary to elucidate what are the molecular mechanisms through which the Scorpins contribute to the DDR. As of now, we still do not know how RANBP9 and RANBP10 protect cells from genotoxic stress. In response to DNA damage, Scorpins may act in the context of the CTLH complex or independently. The involvement of the CTLH complex raises the question whether Scorpins determine protein turnover of important players of the DNA repair.

On a more basic level, the clinical relevance of Scorpins expression/protein levels still requires extensive studies. The lack of validated reagents, particularly anti-RANBP10 specific antibodies, has severely limited the investigation of the relevance of these proteins in the clinical setting. For example, it is not yet possible to establish how well the levels of mRNA correlate with the Scorpin protein amounts. The generation of cell and mouse models by the use of the latest technologies will be necessary to answer many of the open questions. Likewise, a holistic proteomics approach will be essential in establishing how Scorpins through their multiple interactions coordinate the intricate cellular networks responsible for DNA repair, cell cycle arrest, disposal of potentially damaged organelles or protein aggregates upon DDR activation and cell death.

Finally, since Scorpins are involved in responding to different types of stress, from a clinical perspective, it would be interesting whether their absence can sensitize tumors to hyperthermia, for example. In fact, hyperthermia has been shown to sensitize tumors to genotoxic treatments and several DDR players are sensitive to temperature increases [70,71,72].

In summary, the investigation on the role of Scorpins in the cellular DDR is only at initial phases and it is necessary to establish the molecular details of RANBP9 and RANBP10 in determining the most appropriate response to damage of the DNA and whether they can be considered as a viable target for cancer treatment.

## Figures and Tables

**Table 1:** Phosphorylation sites by ATM, ATR and DNA-PK (PIK kinases) on RANBP9 and RANBP10. Selected list of serines and threonines (Amino acids “AA” column) and target peptides predicted by the algorithm Group-Based Prediction System, GPS 3.0 (http://gps.biocuckoo.org) to be phosphorylated by PIK-kinases on RANBP9 (Protein ID: Uniprot Q96S59) and RANBP10 (Protein ID: Uniprot Q6VN20). Protein domains in which the listed target sites are located are also indicated. Selection of predicted sites was based on a score > 2 and a difference between the score and the threshold >20% of the total score. The only exceptions to these criteria are RANBP9 S483 and RANBP10 S365, whose existence is reported in 58 and 160 datasets, respectively from the “PhosphoSitePlus” website. PhosphoSitePlus column lists the number of datasets in which the specific phosphorylation has been reported. For the references associated with those datasets and criteria used for the selection, please visit: www.phosphositeplus.org.

	Protein Domain	Residue	AA	Target Peptide	PhosphoSitePlus
RANBP9 (Uniprot Q96S59)	PRY	181	S	KFSYIGLSQNNLRVH	1
	LisH	375	S	MIQKMVSSYLVHHGY	-
	CTLH	426	T	MGEAIETTQQLYPSL	-
	Post-CTLH Region	470	S	LGGRSPKSQDSYPVS	2
		483	S	VSPRPFSSPSMSPSH	58
		550	S	NSINMSRSQQVNNFT	1
		585	S	NGFLNGSSKHDHEME	-
		603	S	TEMEVDSSQLRRQLC	2
		613	S	RRQLCGGSQAAIERM	2
	CRA	631	S	GRELQAMSEQLRRDC	-
		705	T	ALAMGQATQCLGLMA	-
RANBP10 (Uniprot Q6VN20)	PRY	69	S	KYNYIGLSQGNLRVH	1
	LisH	263	S	VLQNMVSSYLVHHGY	-
	CTLH	314	T	VGEAIETTQRFYPGL	-
	Post-CTLH Region	358	S	LSSRSPKSQDSYPGS	5
		365	S	SQDSYPGSPSLSPRH	160
		377	S	PRHGPSSSHMHNTGA	-
		386	S	MHNTGADSPSCSNGV	7
		439	S	NSTDSTKSQHHSSTS	-
		490	S	DLQTDESSMDDRHPR	13
	CRA	579	S	LNSAILESQNLPKQP	-
